# Newborn screening for Fabry disease in the north-west of Spain

**DOI:** 10.1007/s00431-017-2950-8

**Published:** 2017-06-23

**Authors:** Cristobal Colon, Saida Ortolano, Cristina Melcon-Crespo, Jose V. Alvarez, Olalla E. Lopez-Suarez, Maria L. Couce, José R. Fernández-Lorenzo

**Affiliations:** 1 Unit of Diagnosis and Treatment of Congenital Metabolic Diseases, Complexo Hospitalario Universitario de Santiago de Compostela, Santiago de Compostela, Spain; 2 Rare Diseases & Pediatric Medicine Research Group, Galicia Sur Health Research Institute (IIS Galicia Sur), SERGAS-UVIGO, Hospital Álvaro Cunqueiro, Bloque técnico, pl2 zona A, Estrada Clara Campoamor 341, Vigo, 36312 Pontevedra Spain; 3 Pediatrics Department, Xerencia de Xestión Integrada de Vigo, SERGAS, Vigo, Spain

**Keywords:** Fabry disease, Newborn screening, Lysosomal storage diseases, Genetic variants of unknown significance

## Abstract

Fabry disease is an X-linked lysosomal storage disorder caused by the impairment of α-galactosidase A. Enzyme replacement therapy is available to treat patients, who often experience delayed diagnosis. A newborn screening for Fabry disease was performed to study the prevalence of the pathology and to evaluate the possibility to implement the test in systematic screenings. We collected 14,600 dried blood spot samples (7575 males and 7025 females) and carried out a diagnostic study by fluorometric measurement of α-galactosidase A enzymatic activity and *GLA* gene sequencing. We detected one patient with a mutation in *GLA* associated with classical Fabry Disease (M290I), ten subjects carrying genetic variants of uncertain diagnosis (S126G, R118C, A143T), and a girl with the non-characterized variant F18Y, which was not previously described. Additional 25 samples presented nucleotide substitutions described as polymorphisms (D313Y, rs2071225, and rs2071397). The estimated prevalence for Fabry disease in north-western Spanish males is of 0.013%.

*Conclusion*: These results confirm that the prevalence of Fabry disease is underestimated and systematic screening is feasible; however, further characterization of variants of uncertain clinical significance is necessary to establish protocols of patients’ management.

**Table:** 

**What is Known:** • *Fabry disease is a rare disease of delayed diagnosis, whose prevalence is underestimated. However, early diagnosis is important for better efficiency of the current available treatment.*
**What is New:** • *This newborn screening for Fabry disease performed on Spanish population reveals a prevalence of genetic alterations in GLA of 0.1% in males (0.013% with classic Fabry disease) and also characterizes these modifications in order to discriminate between pathogenic mutations and genetic variants of unknown significance.*

## Introduction

Lysosomal storage disorders (LSDs) are good candidates for newborn screening (NBS) since there are diagnostic methods and treatments available [1, 2]. Indeed, Pompe disease and mucopolysaccharidosis I (MPS I) were included by the Advisory Committee on Hereditable Disorders in Newborns and Children (ACHDNC) in the recommended uniform screening panels (RUSP). This board, which was created by the US Department of Health and Human Services to harmonize NBS panels and methodologies, also evaluated the suitability of early detection for other LSDs, such as Krabbe disease or Fabry disease (FD, OMIM#301500) [3, 4].

In FD, NBS has questionable benefits since the onset of the pathology occurs in adult age and available treatments present important limitations (i.e., low half-life, bioavailability, and compliance, as well as possible immunogenicity). Furthermore, the benefits of treating it since early childhood are still uncertain [5, 6].

An additional point of discussion is the frequent detection of variants in the *GLA* gene (NC_000023.1, mRNA NM_000169.2) with unclear diagnostic interpretation (i.e., p.A143T, p.R118C, p.E66Q…), which complicates the management of subjects identified in FD screenings. These variants have been related to a late-onset phenotype, and patients are occasionally treated with enzyme replacement therapy [7, 8].

Due to these controversial issues, ACHDNC rejected to include FD in RUSP in their first evaluation; however, local laws supported by newborn screening advocates and parents allowed the implementation of systematic screening for FD in Illinois and Missouri, which was also facilitated by the development of cost-sustainable protocols. Pilot studies have also been performed in other states, such as New Jersey and New Mexico, among others [9]. Currently, the discussion on including FD in the RUSP is re-opened due to the development of more compliant treatments, based on orally administering pharmaceutical chaperones. Migalastat (Galafold® Amicus therapeutics) is up to date the only pharmacological chaperon for FD, which obtained the European Medicine Agency (EMA) approval for commercialization [10].

The aim of this study is to estimate the prevalence of FD in the population of Galicia (north-west of Spain) and to evaluate whether it is feasible to include this disease in the NBS program implemented by the public healthcare system of our region, which is currently detecting over 20 metabolic diseases.

## Materials and methods

### Aim and design of the study

A newborn screening for Fabry disease was performed to study the real prevalence of the pathology in our region and to evaluate the possibility to implement the test in systematic screenings. We carried out a diagnostic study based on enzymatic screening of α-galactosidase A.

### Subjects

The enzymatic screening was performed in asymptomatic newborns by collecting dried blood spots (DBSs) from 14,600 individuals (7575 males and 7025 females) who represent 99% of all births in Galicia during the year 2008. Although enzymatic tests are not reliable in screening females, we decided to perform the analysis also in females to estimate if prevalence in males was significantly different from the prevalence in the whole population. This would provide an indirect measure of the number of females that the enzymatic test fails to detect.

### Protocol

Samples were gathered between day 3 and 5 after birth. Clinical history of the subjects was consulted by authorized physicians through the Galician Healthcare System Clinical History Database (IANUS) in order to collect symptoms related to the disease, birth weight, and gestational age. The study was approved by the Healthcare Ethics Committee of Santiago de Compostela University Hospital. Two independent measurements were performed in samples which present lower activity than the cutoff. Informed consent was asked to patients with confirmed low levels of enzymatic activity before performing *GLA* sequencing. A genetic study was also performed to the patient’s parents of this group upon consent.

### Measurements

Enzymatic activity of α-galactosidase A (α-GalA) was measured following the fluorometric method described by Chamoles [11]. Briefly, the assay was performed on triplicates in buffer citrate-phosphate 0.15 M pH 4.2, using 4-methylumbeliferil-galactopiranoside substrate (2 mM, Glycosinth, UK) in the presence of *N*-acetylgalactosamine (70 mM, Sigma-Aldrich). α-GalA activity was expressed as micromoles of substrate per hour and liter of blood (μmol/Lh).

For genetic study, the seven exons of *GLA* were amplified by PCR with specific primers and were sequenced by the Sanger method, using the Chromas 2.4. software to detect point mutations and small deletions or insertions in exons and near intronic regions (±25 bp).

### Data analysis

Correlation of enzymatic activity with birth weight and gestational age was analyzed using the Pearson correlation coefficients. The association between enzymatic activity and gestational age (<35 vs. >35 weeks) or low birth weight (<2500 g) was tested using *χ* ^2^ test for contingency tables. The cutoff value for enzymatic activity was determined using the 0.5 percentile and was adjusted to take into account correlating variables like sex, weight, and gestational age. To establish the appropriate cutoff value, we quantify the α-GalA activity in a total of 2168 healthy newborns. The average activity was 5.1 μmol/Lh (CI 95% 5.0–5.2) and the median was 4.57 (95% CI 4.46 to 4.71). Prevalence, positive predictive value (PPV), and method accuracy for the diagnostic protocol were calculated using the MedCalc 16.4.3 software (Oostende University, Belgium); CI was indicated.

## Results

To identify FD positive subjects, we determined a cutoff value of 1.7 μmol/Lh applying the 0.5 percentile to the cohort of enzymatic activity data; however, this reference was raised at 2 μmol/Lh so that we could take into account correlating variables and avoid false negatives.

Indeed, heterozygous girls have higher residual activity compared to boys, and we calculated that α-GalA activity also correlates with birth weight (*r* = −0.2468, *p* < 0.0001, 95% CI = −0.3027 to −0.1892, *F*-ratio = 68.1874, *p* < 0.001) and gestational age (*r* = −0.3026 *p* < 0.0001, 95% CI = −0.3581 to −0.2450, *F*-ratio = 99.9011, *p* < 0.001). Pre-term babies (<35 weeks gestation) and low birth weight newborns (<2500 g) present higher enzyme levels than term infants (9.1 versus 4.9, *t* = 10.721, DF = 991, *p* < 0.0001) and normal weight children (7.0 versus 4.9, *t* = 8.499, DF = 1051, *p* < 0.0001).

**Fig. 1:**
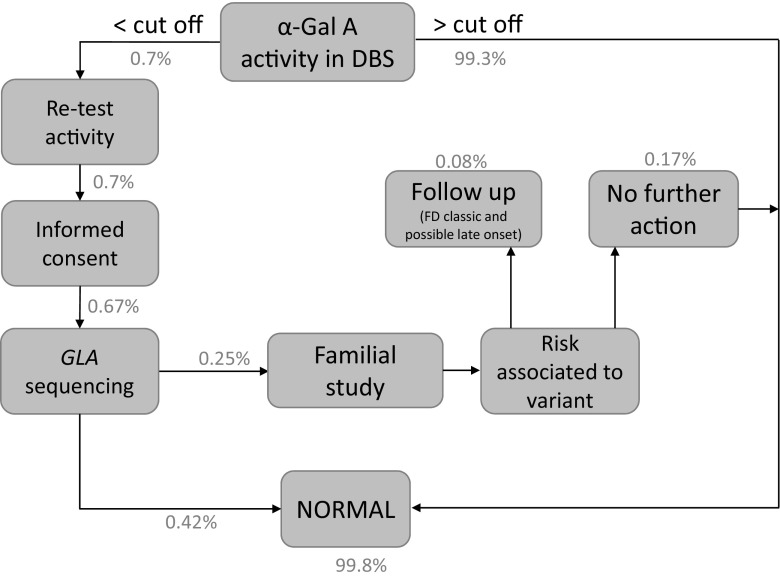
A description of the FD screening protocol. Percentage of subjects (males and females) identified in each step are indicated

Following flourimetric tests, 14,494 samples presented α-GalA activity ≥2 μmol/Lh, while enzymatic levels in 106 samples were lower than the cutoff (Fig. 1), as confirmed by a second measurement. Among patients with enzymatic activity below ≥2 μmol/Lh, we obtained consent for *GLA* sequencing in 101 subjects (68 males and 33 females), while the remaining 5 (females) were excluded from the analysis.

Genetic variants in *GLA* were detected in 37 patients (20 males and 17 females) as summarized in Table 1.

**Table 1:** α-GalA activity levels obtained in two independent biochemical analyses and mutations detected by genetic sequencing. Female heterozygous patients are indicated in italics. The classical FD patient was highlighted with bold font

Patient	Sex	Activity 1st test (μmol/Lh)	Activity 2nd test (μmol/Lh)	Genetic variant
**1**	**M**	**0.6**	**0.6**	**M290I, rs2071397, rs2071225**
*2*	*F*	*1.6*	*1.6*	*F18Y/wt*
*3*	*F*	*2.0*	*1.5*	*S126G*, *rs2071397/wt*
4	M	1.7	1.4	A143T
*5*	*F*	*1.7*	*1.9*	*A143T/wt*
6	M	0.7	1.1	A143T
7	M	1.7	1.3	R118C
*8*	*F*	*1.4*	*1.6*	*R118C/wt*
9	M	1.1	1.5	R118C
10	M	1.4	1.0	R118C
11	M	1.7	1.6	R118C
12	M	2.0	1.8	R118C
*13*	*F*	*1.9*	*1.5*	*D313Y*, *rs2071225*, *rs2071397/wt*
14	M	1.9	1.2	D313Y
15	M	1.8	1.9	D313Y
16	M	1.7	1.6	D313Y
*17*	*F*	*1.9*	*1.7*	*rs2071225/wt*
*18*	*F*	*1.9*	*1.8*	*rs2071225/wt*
19	M	0.6	0.3	rs2071225
20	M	1.4	1.8	rs2071397
*21*	*F*	*1.8*	*1.6*	*rs2071397/wt*
*22*	*F*	*1.5*	*2.0*	*rs2071397/wt*
23	M	1.3	1.1	rs2071397
*24*	*F*	*1.4*	*1.9*	*rs2071397/wt*
*25*	*F*	*1.8*	*1.8*	*rs2071397/wt*
26	M	1.3	1.9	rs2071397
*27*	*F*	*1.6*	*1.8*	*rs2071397/wt*
28	M	1.2	1.8	rs2071225, rs2071397
*29*	*F*	*1.7*	*2.0*	*rs2071225*, *rs2071397/wt*
30	M	1.4	1.5	rs2071225, rs2071397
*31*	*F*	*1.8*	*0.9*	*rs2071225*, *rs2071397/wt*
32	M	1.6	1.8	rs2071225, rs2071397
*33*	*F*	*1.9*	*1.7*	*rs2071225*, *rs2071397/wt*
34	M	1.8	1.6	rs2071225, rs2071397
35	M	1.9	1.9	rs2071225, rs2071397
*36*	*F*	*2.0*	*1.9*	*rs2071225*, *rs2071397/wt*
*37*	*F*	*1.9*	*1.7*	*rs2071225*, *rs2071397/wt*

The p.M290I (c.870G>A) identified in one male patient was previously described as pathogenic and associated to classical FD. Methionine 290 is conserved in at least three α-galactosidase and one β-galactosidase orthologues, and its substitution is related to the misfolding of the protein [12].

The p.F18Y (c.53T>A) missense non-characterized variant was identified in a heterozygous patient, who at the moment does not have a definitive diagnosis of FD. This single nucleotide polymorphism (SNP) was not reported earlier in the Human Mutation Database; [13] however, the database includes as pathogenic a FD-related mutation of phenylalanine 18 with serine. Like serine, tyrosine is more polar than phenylalanine and may alter the hydrophobic interactions of the molecule. A clinical follow-up of the girl, who inherited the mutation from the mother, revealed that she presented dermatological lesions of unidentified cause. She also suffered transitory respiratory impairment and underwent transfontanellar ultrasound, since she presented macrocephalic features, although the exam failed to detect any kind of alteration. At the age of 4, she was also explored in cardiology because extrasystoles were detected. The mother of the index case refused to further collaborate to the study; however, she presented several skin lesions, which have not been studied and the grandfather of the index case died at the age of 32 due to melanoma. Other members of the family (siblings of the mother of the index case, 3 females and 2 males) presented several non-specific symptoms but refused to be tested for FD. One uncle presented angiokeratoma-like lesions and a lesion on the knee which was biopsied. Biopsy examination determined the presence of inflammation and enlarged cells with birefringent-deposited material. One aunt presented recurrent abdominal pain, hearing loss, and occasional pruritus in the extremities.

In the remaining subjects, we detected genetic variants of uncertain significance (GVUS) such as p.R118C (c.352C>T), p.A143T (c.427G>A), and p.S126G (c.376A>G), as well as the pseudodeficiency allele p.D313Y (c.937G>T), and the intronic variants rs2071225 and rs2071397.

Since enzymatic testing is not conclusive for females, we calculated the prevalence of FD and the performance of the diagnostic test considering the data obtained in the male population. The estimated prevalence is of 0.013% (1:7575 males); the PPV of the current screening is 1.47% with a method accuracy of 99.12% (CI 98.9–99.3) and a false positive rate of 0.88%.

## Discussion

The FD prevalence estimated in this study is considerably more frequent than the initially described [14], and the values are comparable to those reported in other studies, which were carried out in different geographical areas. A study conducted in Japanese newborns [15] reported a prevalence of 1:7057 live births with FD classical phenotype, increased to 1:3024, including uncharacterized *GLA* variants. Previous NBS studies in Taiwan and Italy estimated a prevalence of, respectively, 1:2445 live births and 1:3100 males [16, 17], including *GLA* variants of uncertain clinical significance. Van der Tol et al. [18] conducted a systematic review of FD screening studies to recalculate the prevalence of the pathology by discriminating mutations and GVUS and concluded that 90% of the identified cases harbored variants of unclear significance. In European asymptomatic newborns, the most frequent genetic alterations reported p.A143T, p.R112H, and p.D313Y, while in a Taiwanese study, rs2071397 was found in 83% of the patients. Controversial variants were also detected in 84 out of 28,165 patients enrolled in screening studies of high-risk populations (prevalence = 0.3%), in which the classical FD phenotype has a prevalence of 0.12%. Since in screening studies, where GVUS were identified, the selected patients presented cardiac lesion or end-stage renal disease, it is still arguable if these genotypes, which are relatively frequent in the general population (p.A143T = 0.09% and p.R118C = 0.04%), could be directly related to FD and up to date it is recommended not to consider these subjects as patients unless a biopsy examination confirms the presence of deposits [19].

The detection of p.D313Y SNP, which is present in 0.3% of the population [18], is also frequent in previous screening studies; however, there is a general consent in considering this SNP as a pseudodeficiency allele [20].

The nucleotide substitutions in intronic region identified in our study, as well as in previous reports, are validated SNPs [21]. Even if it has been shown that when these SNPs are part of complex haplotypes, they can be involved in *GLA* transcription defects [22, 23], the mechanisms through which transcription is inhibited have not been clarified and these individuals cannot be considered as patients without further evidences.

The most recent NBS studies performed in Washington [24] and Missouri [9] report a FD prevalence of 1:7800 live births (not specifying how many late onset or GVUS) or 1:2913 (10 classic, 4 late onset, and 1 GVUS), respectively. The identified subjects in the Missouri study with *GLA* variants are extensively followed up to determine which individuals actually develop a FD-related phenotype. This follow-up is essential to identify pathogenic and late-onset variants in order to establish a definitive diagnosis.

The second aim of our study was to evaluate the suitability of performing NBS screening for FD in our community by implementing an appropriate method. Although the applied protocol allowed to reach results which are consistent with the ones reported in other literature, the method could be meliorated to reduce false-positive rates (FP = 0.88%) and therefore cost of determinations. Nevertheless, the estimated method accuracy and PPV are comparable with the ones obtained, using the same samples for the determination of immunoreactive trypsinogen (PPV = 4%, method accuracy = 99.2%) and thyroid stimulating hormone (PPV = 18% and method accuracy = 99.8%) by immunofluorometric assays. These tests are routinely carried out in our hospitals for the first step detection of cystic fibrosis (CF) and congenital hypothyroidism using DBS. However, the protocol we apply for CF and congenital hypothyroidism screening requires a second step analysis for those samples that present indeterminate values (close to cutoff points). For CF detection, samples are directed to DNA analysis by mass spectrometry (Maldi-Tof), using a chip with 277 mutations. In the same fashion, a second step analysis based on the quantification by mass spectrometry of the enzymatic reaction products [25] could help to reduce false-positive rates in the screening for FD.

Lowering the cutoff level that we established for α-GalA may also help to reduce false-positive rates of the enzymatic screening, in case that the test is performed in male samples only. The cutoff value of enzymatic activity could have been overestimated in male samples, since it was determined in 2168 healthy newborns of both sexes.

On the other hand, performing enzymatic test in newborn females may help to the early identification of at least a percentage of the affected females. In our study, we detected a heterozygous subject with a new non-characterized variant of *GLA* (p.F18Y), as well as two newborn girls with GVUS. In the case that further studies confirm a pathogenic state of p.F18Y, the prevalence of FD in the whole population will be of 0.014% (1:7297; PPV of 2%, a method accuracy of 99.3%, a false-positive rate of 0.67%, and a 95% CI of 75–100), which are values similar to the ones obtained in male population. This suggests that the percentage of false negatives in our female population is not extremely high. Nonetheless, genetic sequencing is the only method that currently is efficient for a reliable detection of female patients and we have to be well aware that an activity above the cutoff does not exclude FD in females.

When considering newborn screening for FD, there are important issues to take into account. Given the refusal of ACHDNC to include FD in the RUSP, experts maintain an open position regarding NBS for FD or Gaucher disease, which meets favorable criteria in terms of frequency of the diseases and available diagnostic methods and therapies [26]. Even if the ambiguity regarding the prognosis of the subject with GVUS is an undesirable outcome in screening, the possibility to establish an early therapeutic protocol counts as a major pro in favor of neonatal FD screening, since the available treatments are more efficient when the therapy is started in the early symptomatic phase. We believe that the benefits of early diagnosis, especially in the case of positive subjects, cannot be underestimated; therefore, we suggest reconsidering the position adopted on FD screening by ACHDNC, based on the growing amount of data appointing to high frequency of *GLA* mutations in the population and the perspective of new therapeutic strategies. This paper largely discusses the characteristics of genetic modifications in order to discriminate between pathogenic mutations and GVUS and can help to develop adequate follow-up programs. Indeed, in order to support a change in the current policy, a follow-up of the subjects identified in pilot studies will be determinant and should be associated to a deeper functional characterization of the controversial variants, which includes transcription analysis and epigenetic data.

## Conclusions

The estimated prevalence for FD classical phenotype in male newborns of Galicia is of 0.013%. These values, as well as the feasibility and efficacy of the proposed method (method accuracy = 99.12%, PPV = 1.47%), postulate FD as a candidate for systematic screening. However, further characterization of the frequently identified variants of *GLA* with uncertain clinical significance is necessary to establish appropriate protocols for patients’ management.

## Abbreviations

ACHDNC: Advisory Committee on Hereditable Disorders in Newborns and Children
CF: Cystic fibrosis
DBS: Dried blood spots
FD: Fabry disease
GVUS: Genetic variants of unknown significance
LSDs: Lysosomal storage disorders
MPS: Mucopolysaccharidosis
NBS: Newborn screening
PPV: Positive predictive value
SNP: Single nucleotide polymorphism
α-GalA: α-Galactosidase A
